# Inflammation from mild COVID-19 results in persistent neurological and behavioral changes in rhesus macaques

**DOI:** 10.21203/rs.3.rs-8159193/v1

**Published:** 2025-12-02

**Authors:** Tomas R Wiche Salinas, Sienna Freeman, Rebecca Richardson, Winni Weng, Muskan Ali, Alex van Schoor, Elise Viox, Kevin Nguyen, James Auger, Richard Ketan, Matthew Gagne, Breanna Picou, Nadia A Golden, Monica Vaccari, Sherri Jean, Jennifer S Wood, Joyce Cohen, R. Paul Johnson, Daniel C Douek, Benoit A Niclou, Rebecca D Levit, Ian N Moore, Mirko Paiardini, Jessica Raper

**Affiliations:** 1 Emory National Primate Research Center, Emory University, 954 Gatewood Rd NE, Atlanta GA 30329, USA; 2 Vaccine Research Center, National Institute of Allergy and Infectious Diseases, National Institutes of Health, Convent Drive, Bethesda MD 20892, USA.; 3 Tulane National Biomedical Research Center, Tulane University School of Medicine, 18703 Three Rivers Road, Covington LA 70433, USA.; 4 Department of Medicine, Division of Cardiology, Emory University School of Medicine, 1750 Haygood Drive, Atlanta GA 30322, USA.; 5 Department of Pathology and Laboratory Medicine, Emory University School of Medicine, 1364 Clifton Road, Atlanta GA 30322, USA; 6 Department of Medicine, Division of Infectious Disease, Emory University School of Medicine, 1750 Haygood Drive, Atlanta GA 30322, USA.; 7 Department of Pediatrics, Emory University School of Medicine, 2015 Uppergate Dr, Atlanta GA 30329, USA; 8 Children’s Healthcare of Atlanta, 1575 Northeast Expressway, Atlanta GA 30329, USA

**Keywords:** Nonhuman primate, PASC, Neuroinflammation, Sex differences, Heart rate variability, Long-COVID

## Abstract

Although most SARS-CoV-2 infections result in mild or moderate symptoms not requiring hospitalization, many patients experience persistent symptoms after their initial recovery, a condition termed Post-Acute Sequelae of SARS-CoV-2 infection (PASC). The underlying pathogenesis behind infection associated chronic illnesses, such as PASC, are poorly understood, thus critically limiting the development of therapeutics to prevent or alleviate symptoms. The current study examined the neurocognitive impact of SARS-CoV-2 induced inflammation in a nonhuman primate model. Ten adult rhesus macaques (5 female, 5 male) were monitored before, during, and after recovery from a mild COVID-19 illness (SARS-CoV-2 strain 2019-noCoV/USA-WA1/2020). Macaques exhibited persistent alterations in taste and smell, as well as decreased cognitive flexibility up to 3 months post-infection. Female macaques experienced sleep disturbances, greater stress and poorer autonomic function months after SARS-CoV-2 infection. Importantly, the development of these neurocognitive changes were associated with acute cytokine response to infection and increased microglia activation in brain tissue at 4 months post-infection. These findings suggest a causative link between the inflammatory response to mild COVID-19 symptoms and persistent neurocognitive changes associated with PASC and provide rationale for therapeutic strategies aimed at reducing acute inflammatory responses to the virus.

## One-Sentence Summary

Inflammation induced by mild SARS-CoV-2 infection results in persistent neuroinflammation and neurocognitive changes in a clinically translational nonhuman primate model.

## Introduction

Infection-associated chronic illnesses are characterized by persistent symptoms following an initial infection even though the original pathogen is no longer detectable ^1^. Increased focus on infection-associated chronic illnesses emerged during the COVID-19 pandemic. Most people infected with SARS-CoV-2 experienced mild symptoms, while only ~10–15% of cases progressed to severe illness, and about 5% became critically ill, requiring hospitalization and intensive care ^2^. Yet, a large number of recovered COVID-19 patients (10–40%) present with symptoms weeks or even months after their initial recovery, termed Post-Acute Sequelae of SARS-CoV-2 infection (PASC or Long-COVID), with symptoms including sustained loss of taste or smell, “brain fog”, neuropsychiatric symptoms, fatigue, shortness of breath, sleeping disorders, and gastrointestinal symptoms^2–12^. Similar long-term effects have been described in individuals infected with the original SARS-CoV, where survivors developed impaired exercise capacity^13^ and abnormal pulmonary function that required years to resolve^14^.These persistent symptoms after recovering from COVID-19 are creating a substantial healthcare and economic burden for millions of people^15^.

Despite the awareness of PASC and the rapid and extensive efforts made to study this post-infection chronic illness, patients lack effective treatments due to our poor understanding of the pathogenesis that leads to long-term neurological complications. There is a considerable need to understand how viral replication and inflammatory marker levels, during the acute phase of infection and those persisting months after recovery, may serve as predictors of patient symptoms. Although increased inflammation (e.g. cytokines) markers have been found in both severe and non-hospitalized patients with SARS-CoV-2,^16,17^ due to ethical reasons, there is very limited data regarding how mild SARS-CoV-2 infections directly impact the central nervous system (CNS) of patients. Therefore, an animal model that mimics the virological, immunological, and neurological features of SARS-CoV-2 infection in humans would provide an essential opportunity to directly examine the long-term impact of SARS-CoV-2 infection on the brain; thus expediting the development, testing, and implementation of future therapeutic strategies.

The immune systems and complex neuroanatomy of nonhuman primates are very similar to humans, making nonhuman primates a valuable translational model for investigating the neurological impacts of SARS-CoV-2 infection^18–26^. In this study, we aimed to establish whether acute inflammation induced during mild SARS-CoV-2 infection (experienced by the majority of infected people) resulted in PASC-like symptoms and measurable neurocognitive changes in adult rhesus macaques (RMs), as well as whether those changes correspond with persistent neuroinflammation in postmortem brain tissue.

## Results:

### Long-term SARS-CoV-2 Kinetics in Rhesus Macaques:

**Figure 1.**
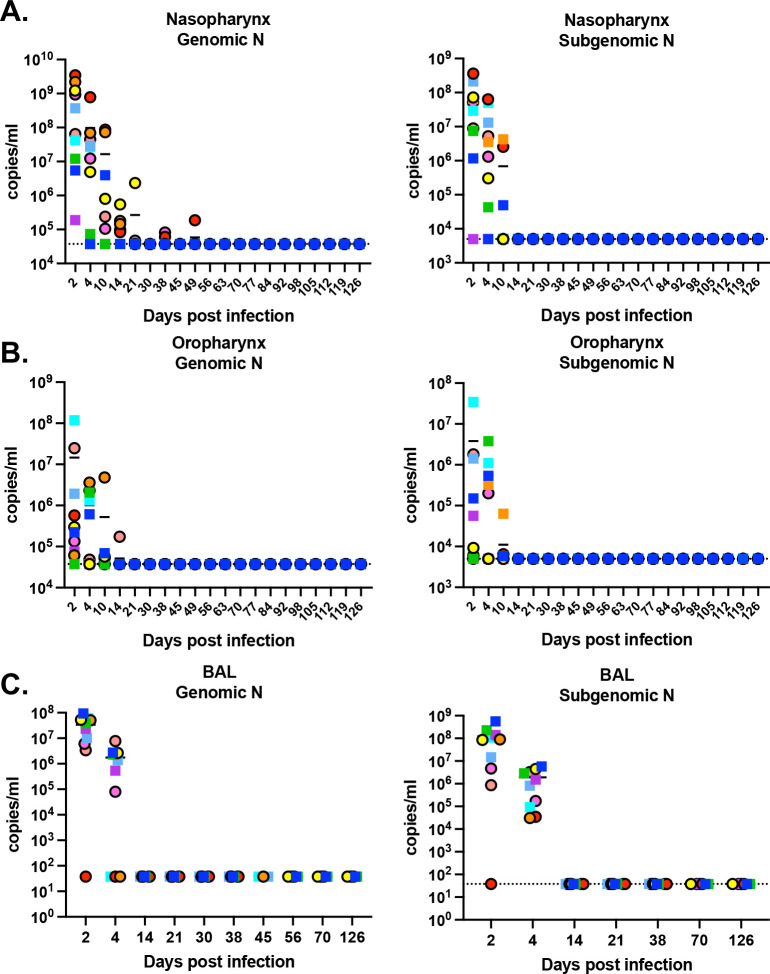
Long-term SARS-CoV-2 kinetics in RMs. Most RMs cleared the virus within 2–3 weeks, as shown by genomic levels measured in in nasopharynx swabs(A), oropharynx swabs(B), and bronchoalveolar lavage (C). Females are represented by circles and males are represented by squares.

Ten adult rhesus macaques (RMs; 5 females and 5 males, aged 6–20 years) were inoculated intranasally and intratracheally with the SARS-CoV-2 strain 2019-nCoV/USA-WA1/2020 at a dose of 1.1 × 10^6^ PFU. Viral RNA loads were monitored longitudinally using quantitative reverse transcription polymerase chain reaction (qRT-PCR), measuring both genomic RNA (gRNA) and subgenomic RNA (sgRNA) of the N gene of SARS-CoV-2 in nasal and oropharyngeal swabs, as well as bronchoalveolar lavage (BAL). The study followed RMs for up to 126 days post-infection (dpi). gRNA and sgRNA N viral loads peaked 2–4 dpi in the nasopharynx (Figure 1A), oropharynx (Figure 1B), and BAL (Figure 1C). However, one RM (RM8) exhibited peak gRNA N levels at 10 dpi, and another RM (RM4) showed peak sgRNA N levels at 10 dpi in the oropharynx (Figure 1B). Viral clearance dynamics varied across the nasopharynx, oropharynx, and BAL. gRNA and sgRNA N in BAL (Figure 1C), as well as sgRNA N in the nasopharynx (Figure 1A, right panel) and oropharynx (Figure 1B, right panel), were cleared by 14 dpi, and gRNA N in the nasopharynx was cleared by 30 dpi in all animals. Notably, two animals exhibited viral blips on 38 dpi (RM3 and RM4), and one animal showed a blip on 49 dpi (RM4).

### SARS-CoV-2 long term persistence in RMs:

**Figure 2.**
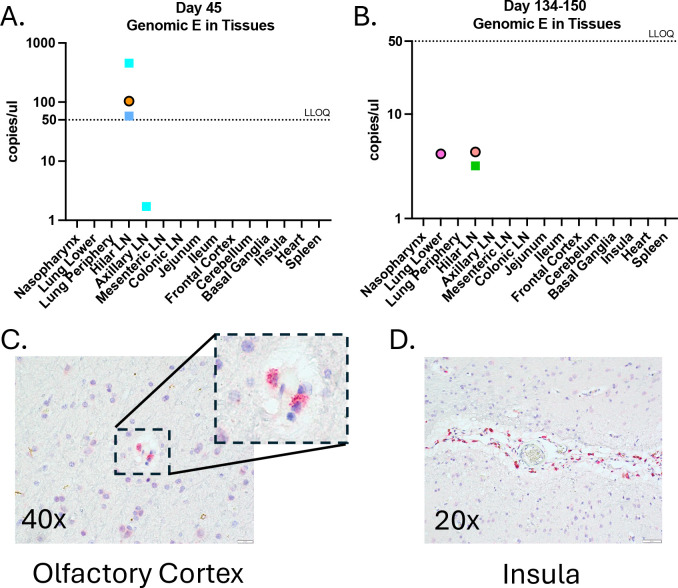
Long-term SARS-CoV-2 persistence in tissues in RMs. Genomic levels of SARS-CoV-2 were detectable above the LLOQ in the hilar lymph node **(A)** at 45 dpi, and very low levels below the LLOQ were detected between 134 and 150 dpi **(B)**. Viral antigens were identified in brain tissues at 45 dpi, specifically in the olfactory cortex **(C)** and the insula **(D)**. Females are represented by circles, and males are represented by squares.

To investigate the long-term persistence of SARS-CoV-2 in tissues, we evaluated the presence of E gene gRNA and the nucleocapsid protein in multiple tissues. This analysis was performed at 45 dpi in three animals (1 female: RM8, 2 males: RM9 and RM10), and between 134 and 150 dpi in the remaining seven animals (4 females, 3 males). At 45 dpi, gRNA E levels above the lower limit of quantification (LLOQ) were detected in the hilar lymph nodes (LNs) of all three RMs. In one RM male (RM9), gRNA E was detected in the axillary LNs at levels below the LLOQ but still present (Figure 2A). Between 134 and 150 dpi, gRNA E was again detected in the hilar LNs of one female (RM2) and one male (RM5) RMs, with levels below the LLOQ but consistently present, similar to the findings at 45 dpi. Additionally, traces of gRNA E were also observed in the lung of one RM female (RM3) (Figure 2B). Furthermore, we assessed the presence of SARS-CoV-2 nucleocapsid protein in multiple tissues in all animals at 45 dpi and between 134 and 150 dpi. Nucleocapsid protein was detected in one RM female (RM8) at 45 dpi in the olfactory cortex and insula (Figure 2C–D). These findings align with reports of long-term, low levels SARS-CoV-2 persistence in human tissues.

### Cytokine and cellular immune profiling in BAL following SARS-CoV-2 infection:

**Figure 3.**
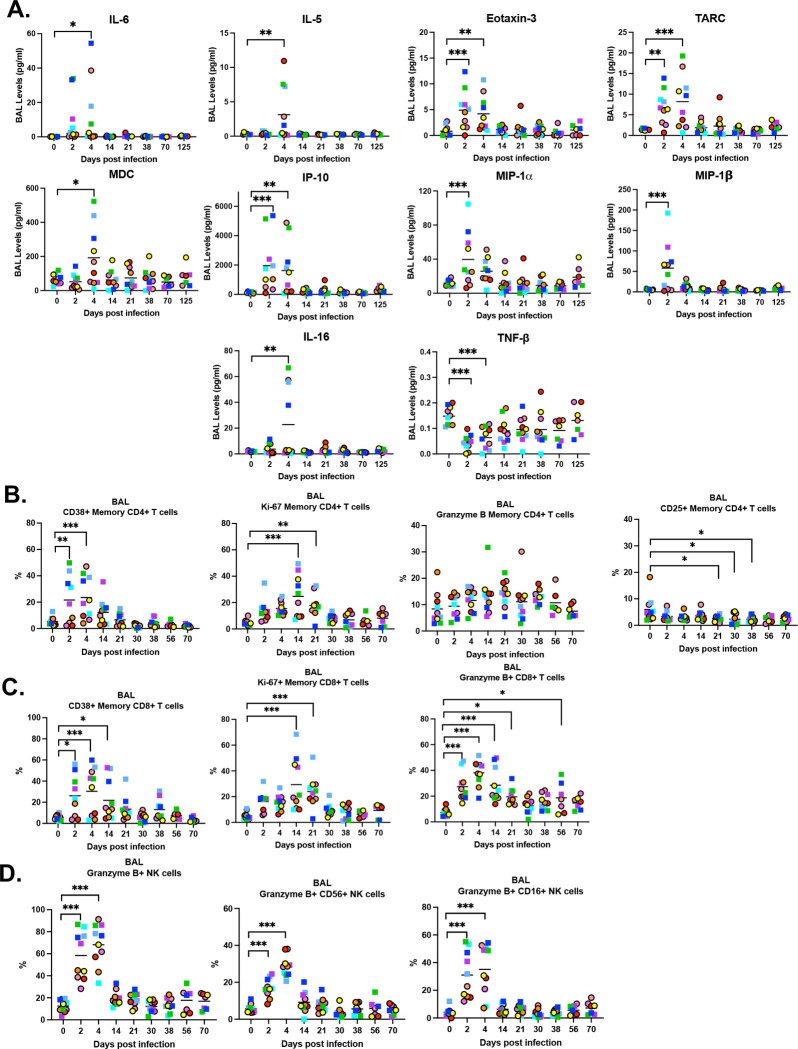
Cytokine and cellular immune profiling in BAL following SARS-CoV-2 infection. Cytokines and chemokines were evaluated longitudinally in BAL fluid using the Meso Scale Discovery (MSD) immunoassay (A). CD4+ T cells (B), CD8+ T cells(C), and NK cells(D) from BALs were phenotypically examined longitudinally by flow cytometry. Females are represented by circles, and males are represented by squares.

To evaluate lung inflammation after SARS-CoV-2 infection and determine its duration, we performed an extensive longitudinal cytokine and cellular profiling analysis in BAL. BAL samples were analyzed for cytokines by mesoscale assay at baseline and at 2, 4, 14, 21, 38, 70, and 125 dpi (Figure 3A), whereas cellular profiling was performed by flow cytometry at baseline and at 2, 4, 14, 21, 30, 38, 56, and 70 dpi (Figure 3B-D).

For most upregulated cytokines we observed a peak at 2 or 4 dpi, with no significant differences at later time points compared with baseline. The upregulated markers included pro-inflammatory cytokines (e.g. IL-6), Th2-associated cytokines and chemokines (e.g., IL-5, Eotaxin-3, TARC, MDC), interferon-induced responses (e.g., IP-10), and chemokines involved in the migration of pro-inflammatory leukocytes (e.g., MIP-1α, MIP-1β, IL-16). In contrast, TNF-β levels were lower at 2 and 4 dpi compared with baseline (Figure 3A).

We also evaluated activation (CD38), proliferation (Ki-67), and cytotoxic capacity of memory CD4^+^ (Figure 3B) and CD8^+^ (Figure 3C) T cells. CD4^+^ T cells showed a significant increase in activation at 2 and 4 dpi, whereas CD8^+^ T cells displayed increased activation up to 14 dpi. A significant increase in proliferation was observed at later time points (14 and 21 dpi) in CD4+ and CD8+ T cells. Granzyme B expression was elevated only in CD8^+^ T cells, with increases at 2, 4, 14, 21, and 56 dpi.

Finally, we assessed the cytotoxic (Granzyme B+) capacity of NK cells (Figure 3D). We observed an early increased granzyme B expression in both total NK cells and CD56^+^ and CD16^+^ subsets at 2 and 4 dpi, which subsequently returned to baseline levels.

### Systemic cytokine, metabolic, and cellular immune responses after SARS-CoV-2 infection:

**Figure 4.**
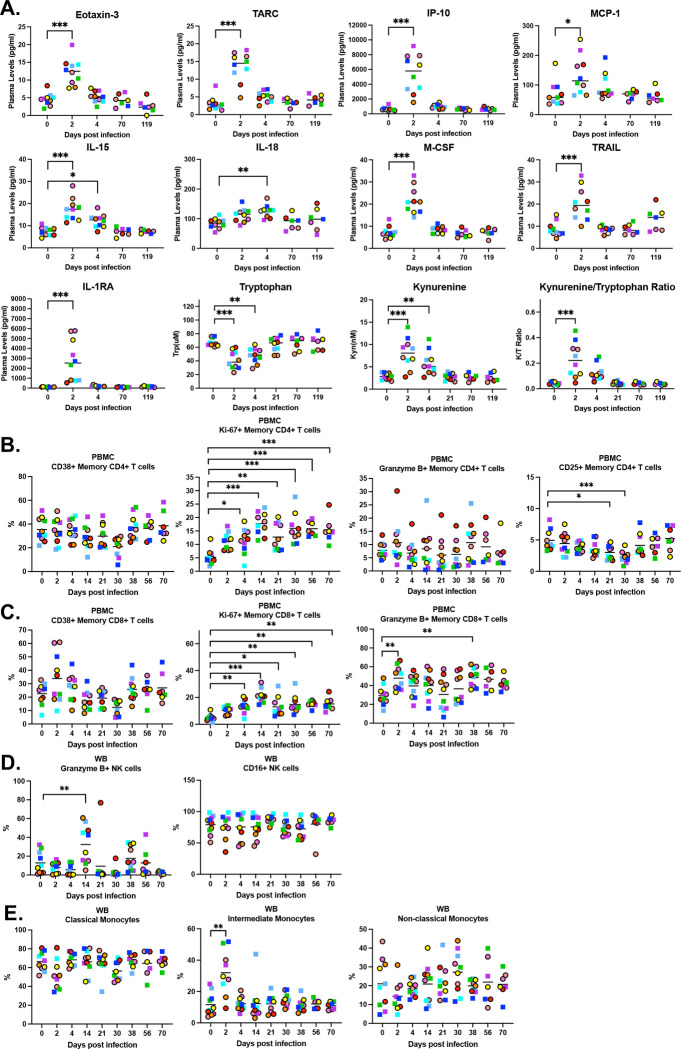
Systemic cytokine, metabolic, and cellular immune responses after SARS-CoV-2 infection. Cytokines, chemokines, and metabolites were evaluated longitudinally in plasma (A). CD4^+^ T cells (B), CD8^+^ T cells (C), and NK cells (D) from PBMCs were phenotypically examined over time by flow cytometry. Females are represented by circles, and males are represented by squares.

To evaluate systemic immune responses after SARS-CoV-2 infection and determine their trajectory overtime, we performed longitudinal cytokine and metabolite analysis in plasma (Figure 4A) and cellular profiling in PBMCs (Figure 4B-E). Plasma was analyzed at baseline and at 2, 4, 70, and 119 dpi, whereas PBMCs were assessed by flow cytometry at 2, 4, 14, 21, 30, 56, and 70 dpi.

For cytokines that increased after infection, we observed a peak at 2 or 4 dpi, with no significant differences at later time points compared with baseline (Figure 4A). Elevated cytokines included Th2-associated mediators (Eotaxin-3, TARC), interferon-induced responses (IP-10), and monocyte-associated factors (MCP-1, M-CSF). Additional increases were detected in the common γ-chain cytokine (IL-15), the inflammasome-related cytokine (IL-18), the cell death receptor ligand (TRAIL), and the anti-inflammatory cytokine (IL-1RA). Metabolic analysis revealed a reduction in tryptophan and a concomitant increase in kynurenine at 2 and 4 dpi, resulting in a significantly elevated kynurenine/tryptophan ratio at 2 dpi. All cytokine and metabolite levels had returned to baseline values by 70 and 119 dpi.

Flow cytometry analysis of PBMCs showed that neither circulating CD4^+^ T cells nor CD8^+^ T cells exhibited an increase in activation (CD38^+^); however, there was a progressively enhanced proliferation (Ki-67^+^) from 4 to 70 dpi. CD8^+^ T cells showed increased Granzyme B expression at 2 and 38 dpi. Finally, CD25^+^ regulatory CD4^+^ T cells decreased at 21 and 30 dpi. (Figure 4B–C). NK cells displayed an increase in cytotoxic capacity (Granzyme B^+^) at 14 dpi, with no differences in CD16 expression (Figure 4D). As we have previously demonstrated, we observed an increase in intermediate monocytes, whereas classical and non-classical monocyte subsets remained unchanged (Figure 4E). Intermediate monocytes that were upregulated expressed the chemokine receptor CCR2, the adhesion molecule CD11b, the IFN-I–inducible receptor sialic acid-binding Ig-like lectin 1 (Siglec-1/CD169), as well as the scavenger receptor CD163 (Supplemental Figure 2).

### Neuroinflammatory responses in the cerebrospinal fluid (CSF) and brain following SARS-CoV-2 infection:

**Figure 5.**
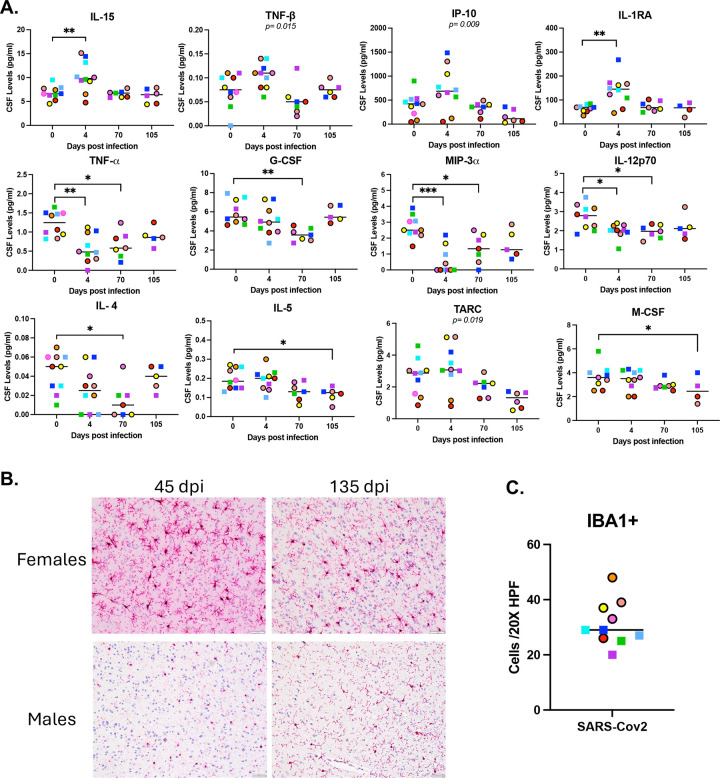
Neuroinflammatory responses in the cerebrospinal fluid (CSF) and brain following SARS-CoV-2 infection. Cytokine and chemokine levels were longitudinally measured in CSF (A). Staining for IBA1 in the insula of female and male RMs following SARS-CoV-2 infection at 45 dpi or 135 dpi (B). All images are 20X magnification and scale bar represents 50 microns. IBA1+ cell counts for each individual animal (C), circles represent females and squares represent males.

To evaluate if inflammation was present also in the central nervous system, we evaluated cytokines and chemokines in the cerebrospinal fluid at baseline, and at 4, 70, and 105 dpi (Figure 5A). We observed an increase of pro-inflammatory cytokines (IL-15, TNF-beta) and interferon-induced response (IP-10) as well as compensatory anti-inflammatory protein IL-1RA at 4 dpi. In contrast to what we observed in BAL and plasma, we observed a decrease in pro-inflammatory cytokines (TNF-alpha), Th1 cytokines (IL-12p70), growth factors (G-CSF), and chemokines (MIP3-alpha) at 4 dpi that persisted through 70 dpi. Th2 cytokines (IL-4, IL-5, and TARC) and growth factor (M-CSF) also showed a late drop at 70 and 105 dpi.

Finally, we used the pan-microglial marker IBA1 (ionized calcium-binding adaptor molecule 1) to evaluate brain sections for neuroinflammatory changes following SARS-CoV-2 infection (Figure 5B, Supplementary Figure 3). A range of IBA1 expression levels was observed, indicative of differing degrees of inflammatory change at 45 and 135 dpi (Figure 5C). While males appear to have fewer IBA1 cells compared to females, the morphology of microglia in males tend to be amoeboid (Figure 5B & 5C).

### Persistent changes in taste and smell:

**Figure 6.**
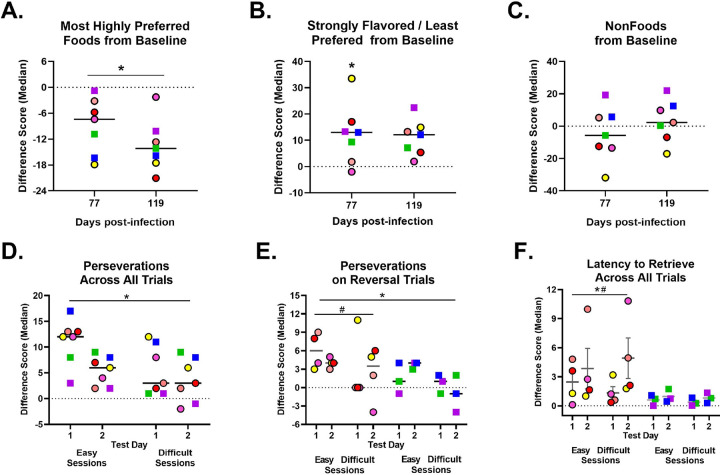
Neurocognitive changes after SARS-CoV-2 infection. Difference score from baseline (dotted line) shows a decreased preference for the top 5 most highly preferred food at 77dpi and 119dpi (A). Increased preference for foods with strong flavors that were least preferred at 77dpi, but no difference at 119dpi (B). SARS-CoV-2 infection did not change preference for nonfood items (C). Difficulty with flexible decision making is shown by an increased number of perseverative reaches across all trials (D), an increased number of perseverative reaches on reversal trials only (E) at 85dpi. Females, but not males, exhibited an increase latency to retrieve the reward 85dpi (F). Individual RMs are represented by different colors. Females are represented by circles with black outlines with dark grey line depicting the median. Males are represented by squares with a light grey line depicting the median. A black line indicates the median for both males and females combined (panels A-D). * indicates a significant difference from baseline, whereas # indicates a significant sex difference p < 0.05.

Loss or alterations in smell and taste are common in SARS-CoV-2 infected humans^27^ and anosmia has been detected in rodent models after SARS-CoV-2 infection.^28,29^ To examine whether RMs exhibited similar changes in taste and smell, we conducted a Food Preference Task at baseline, 77, and 119 dpi. Preference for the top 5 most highly preferred foods was significantly diminished after SARS-CoV-2 infection (Time effect: p <0.001). This decreased preference from baseline for their most highly preferred foods was found at 77dpi (p = 0.007) and persisted through 119 dpi (<0.001; Figure 6A). While preference decreased for the most palatable foods after SARS-CoV-2 infection, there was an increased preference for the foods that had the strongest flavor and were least preferred at baseline (Time effect: p = 0.037; Figure 6B), which was statistically significant at 77dpi (p = 0.040) but not at 119dpi (p = 0.067). There was no difference in preference for nonfood items after SARS-CoV2 infection (Time effect: p = 0.456; Figure 6C). These data suggests that RMs experience changes in taste and smell, as shown by their persistent decreased preference for highly preferred foods up to ~4 months post-infection. Furthermore, the increased preference for strongly flavored foods (e.g. garlic) post-infection suggest the RMs are experiencing a persistent blunted taste and smell compared to baseline.

### Decreased cognitive flexibility:

After SARS-CoV-2 infection, patients commonly report experiencing “brain fog”^30,31^, which can be defined as an inability to focus and lack of cognitive flexibility that leads to a deficit in executive functioning and memory impairment. In RMs we can use the Detour Task ^32,33^ to investigate cognitive flexibility by measuring the animal’s ability to adjust their behavior according to changes in their environment. More specifically, RMs must reach around a barrier to retrieve a food reward, as well as change their detoured reach on reversal trials when the barrier has been switched. At 85 dpi, RMs exhibited decreased cognitive flexibility, as shown by a significant increase in the number of perseverative reaches across all trials compared to their performance at baseline (Time effect: p = 0.003; Figure 6D). Focusing on reversal trial performance also revealed an increased number of perseverative reaches (Time effect: p = 0.006) with females having more perseverations than males on reversal trials (Sex effect: p = 0.028; Figure 6E). Lastly, only females had longer latencies to retrieve the reward after SARS-CoV-2 infection (Time X Sex interaction: p = 0.034; Figure 6F). Overall, these data suggest that SARS-CoV-2 infection impairs cognitive flexibility at ~3 months post-infection and that females may be more greatly impacted compared to males.

### Changes in circadian rhythm and sleep patterns:

**Figure 7.**
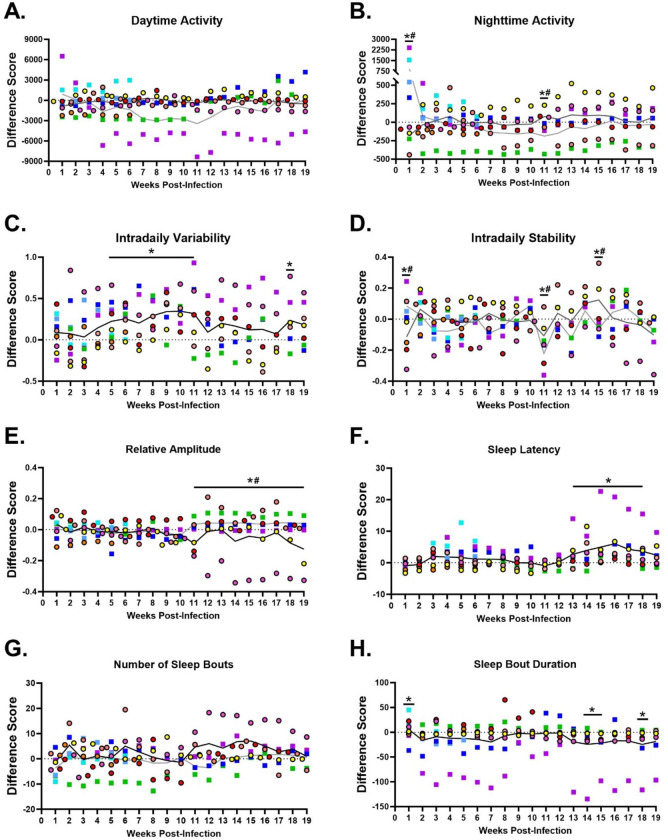
Circadian rhythm changes and sleep disturbances after SARS-CoV-2 infection. Difference score from baseline (dotted line) shows no change in activity during daytime hours (A), yet sex specific changes in nighttime activity (B) at 1 and 11 weeks post-infection (wpi). Increased intradaily variability (C) after infection, but intradaily stability (D) and relative amplitude differed by sex (E). Increased latency to fall asleep (F) was detected months after acute SARS-CoV-2 infection. Despite no significant differences in the overall number of sleep bouts, there were changes in sleep bout duration at 1, 14, 15, and 18 wpi. Individual RMs are represented by different colors. Females are represented by circles with black outlines with dark grey line depicting the mean. Males are represented by squares with a light grey line depicting the mean. A black line indicates the mean for both males and females combined, as shown on panels C, F, and H. * indicates a significant difference from baseline, whereas # indicates a significant sex difference p < 0.05.

Fatigue, post exertional malaise, and sleep disturbances have been reported in patients after SARS-CoV-2 infection.^30,34^ Actigraphy data was collected using an Actical accelerometer placed on a collar that the animal wore throughout the study. Analysis of the activity data revealed no detectable fatigue in the RMs after SARS-CoV-2 infection, as shown by the overall daytime activity counts (Time effect: p = 0.78, Figure 7A). However, sleep disturbances were detected with nighttime activity counts differing between female and male RMs after SARS-CoV-2 infection (Time X Sex: p < 0.001), such that males exhibited greater nighttime activity compared to females during acute infection (1-week post-infection [wpi]; p = 0.05), whereas females were more active overnight at 11 wpi (p = 0.044; Figure 7B). Some features of circadian rhythmicity were altered after SARS-CoV-2 infection. Higher fragmentation of the 24-hour rest-activity rhythm was indicated by increased intradaily variability (IV; Time effect: p = 0.021, Figure 7C) found from 5 to 11 wpi (p = 0.019, 0.008, 0.026, 0.003, 0.002, 0.001, 0.002, respectively) and at 18 wpi (p = 0.035) in both males and females. Sex differences were found for other circadian rhythm features, including intradaily stability (IS; Time X Sex: p = 0.009) and relative amplitude (Time X Sex: p = 0.048). High IS indicates good synchrony to light/dark cycle and female RMs had lower IS during acute infection (1 wpi; p = 0.042), but was higher than males at 11 (p = 0.05) and 15 wpi (p = 0.045; Figure 7D). Lower activity at night and higher activity during the day is an indication of a robust 24-hour rest-activity rhythm, or high relative amplitude. Male RMs had significantly higher relative amplitude compared to females after SARS-CoV-2 infection from 11 to 19 wpi (p = 0.002, 0.022, 0.016, 0.01, 0.01, 0.022, 0.018, 0.017, 0.027, respectively; Figure 7E). There were no significant differences for most active 10-hour period (M10; Time effect: p = 0.72) or least active 5-hour period (L5; Time effect: p = 0.90).

Sleep patterns in RMs were also affected by SARS-CoV-2 infection. Latency to fall asleep increased after infection (Time effect: p < 0.001, Figure 7F) with significantly longer latencies from 13 to 18 wpi compared to baseline (p = 0.035, 0.008, < 0.001, <0.001, 0.001, 0.006, respectively). While SARS-CoV-2 infection did not impact the number of sleep bouts (Time effect: p = 0.37), the average duration of each sleep bout was significantly altered (Time effect: p = 0.014, Figure 7G). During acute infection (1 wpi) animals exhibited longer sleep bouts (p = 0.048), whereas shorter sleep bouts were found at 14 (p = 0.017), 15 (p=0.37) and 18 wpi (p = 0.015, Figure 7H). Taken together the combination of results showing increased nighttime activity, increased IV, lower relative amplitude, longer sleep latency and shorter sleep bouts suggest that female RMs exhibited more sleep disturbances after SARS-CoV-2 infection compared to males.

### Changes in Heart rate Variability:

**Figure 8.**
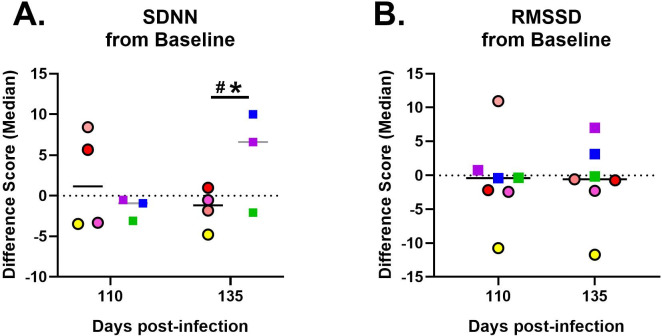
Heart rate variability changes after SARS-CoV-2 infection. Difference score from baseline (dotted line) shows that females and males differed on their heart rate variability SDNN at 135dpi (A), whereas there are no significant changes in RMSSD (B). Females are represented by circles with black outlines with dark grey line depicting the mean. Males are represented by squares with a light grey line depicting the mean. A black line indicates the mean for both males and females combined, as shown on panel B. * indicates a significant difference from baseline, whereas # indicates a significant sex difference p < 0.05.

Cardiac activity, such as heart rate and contractility are modulated by a variety of inputs, including the autonomic nervous system. The variability in heart rate over different time scales is an easily measured reflection of autonomic activity and neurocardiac physiology and is useful in assessing cardiovascular health and predicting disease ^35^. Reduced heart rate variability is associated with diseases including myocardial infarction, heart failure, and orthostatic intolerance syndromes including postural orthostatic tachycardia syndrome (POTS) ^36–38^. To examine heart rate variability after SARS-CoV-2 infection, we collected 5 minute long electrocardiograms (EKG) at baseline, ~110, and ~135 dpi. Results revealed that females exhibited decreased standard deviation of normal-to-normal inter-beat intervals (SDNN) after infection compared to males (Time X Sex: p = 0.047; Figure 8A). SDNN is the heart rate variability parameter most predictive of cardiovascular events and reflects the deviation from mean over the period of the recording. It reflects both sympathetic and parasympathetic nervous system influence. Root mean of squares of successive differences (RMSSD) reflect short term variability under parasympathetic nervous system influence. However, there were no significant changes in the RMSSD (Time: p = 0.97; Figure 8B) of RMs after SARS-CoV-2 infection. Overall, the decreased SDNN among female RMs may indicate greater stress and/or poorer autonomic function after SARS-CoV-2 infection.

### Relationship between inflammatory response and neurocognitive changes after infection:

**Figure 9.**
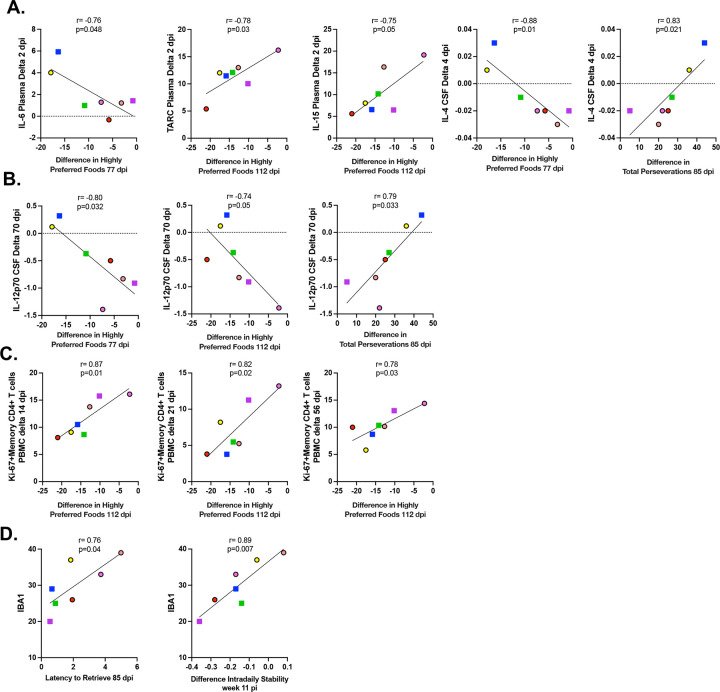
Relationship between inflammatory response and neurocognitive changes after infection. Correlations between behavioral assessments and early (A) and late (B) changes in cytokine levels after SARS-CoV-2 infection in plasma or CSF. Correlations between behavioral evaluations and changes in immune cells after SARS-CoV-2 infection in PBMCs (C) or IBA1^+^ cells in the brain (D). Individual animals are shown in different colors, with circles representing females and squares representing males.

Finally, we correlated immunological changes with neurocognitive outcomes to evaluate potential links between the two features. Specifically, our goal was to identify which early (Figure 9A) or late (Figure 9B) changes in cytokine and chemokine levels, immune cell subset dynamics (Figure 9C), and brain inflammation (Figure 9D) were associated with persistent alterations in taste, smell, or cognitive flexibility.

An increase in plasma IL-6 from day 0 to day 2 (Δday2–day0) negatively correlated with preference for highly preferred foods at 77 dpi, indicating that a greater rise in this pro-inflammatory cytokine during acute infection was associated with more severe and persistent deterioration of taste and smell. An increase in plasma levels of the Th2-associated chemokine TARC and the antiviral protein IL-15 at 2 dpi negatively correlated with the decline in preference for highly preferred foods at 112 dpi, suggesting that early elevations in these cytokines may be protective against deterioration of taste and smell.

An increase in CSF IL-4 from day 0 to day 4 (Δday4–day0) negatively correlated with later food preference, indicating that higher IL-4 levels at 4 dpi were detrimental for preserving taste and smell. In addition, an increase in CSF IL-4 positively correlated with impaired cognitive flexibility, as reflected by increased total perseverations measured at 85 dpi. A decrease in CSF IL-12p70 from day 0 to day 70 (Δday70–day0) negatively correlated with preference for highly preferred foods at 112 dpi and positively correlated with impaired cognitive flexibility at 85 dpi, indicating that higher levels of IL-12p70 are deleterious for both taste/smell and cognitive function. Finally, an increase in CD4+ T cell proliferation at 14, 21, or 56 dpi relative to baseline (Figure 9C) positively correlated with improved olfactory outcomes.

Brain inflammation, evaluated by IBA-1 expression, showed a positive correlation with reduced cognitive flexibility (increased latency to retrieve) and with alterations in circadian rhythm (IS, week 11).

Overall, these results indicate that early immune-inflammatory changes in plasma and CSF after mild SARS-CoV-2 infection are associated with more severe and persistent neurocognitive changes.

## Discussion

Infection-associated chronic illnesses, such as PASC, are characterized by persistent symptoms following an initial infection even though the original pathogen is no longer detectable ^1^. Unfortunately, there is currently no cure for patients, and treatment options remain limited, due to our incomplete understanding of the underlying pathogenesis and immunological perturbations associated with PASC. The current study in adult RMs demonstrated that mild SARS-CoV-2 infection-induced inflammation resulted in persistent PASC-like symptoms for several months after recovering from the initial infection. Importantly, the measurable neurocognitive changes were associated with systemic and nervous system perturbations, indicating a link between infection-induced inflammation and long-term neurological and behavioral outcomes.

The persistence of viral material in tissues in our model is consistent with observations of viral persistence in humans. Residual viral RNA may be intermittently translated, potentially driving sustained immune activation and cellular exhaustion ^18,19,39–42^. Future NHP studies should address whether viral persistence contributes to the neurobehavioral changes observed after SARS-CoV-2 infection.

After SARS-CoV-2 infection, we observed a rapid and robust upregulation of pro-inflammatory cytokines, interferon-induced responses, Th1 and Th2 cytokines, as well as key chemokines involved in leukocyte migration in both BAL and plasma. These findings are consistent with our previous studies and with other non-human primate models aimed at understanding the pathophysiology of long COVID ^18–20,25,26^. Notably, this strong inflammatory response occurs despite the mild nature of the SARS-CoV-2 infection, allowing us to investigate whether early inflammatory changes in mild cases are associated with long-term neurobehavioral outcomes, as detailed in our Results section. This model of long COVID could therefore be used to test interventions designed to reduce inflammation and assess their efficacy in preventing long COVID symptomatology.

Furthermore, we observed transient T cell activation in the BAL, accompanied by more sustained changes in T cell proliferation in the blood. We also detected an early increase in pro-inflammatory intermediate monocytes. Human studies support the hypothesis that T cell dysregulation is associated with long COVID, as well as with the expansion of non-classical monocytes^43,44^. In humans, a reduction in the number of central memory CD4^+^ T cells has been reported, which may be attributed to decreased proliferative capacity. In line with these findings, we observed increased proliferation of memory CD4^+^ T cells in RMs with milders impairments of taste and smell.

Throughout the COVID-19 pandemic, patients commonly reported altered sensory processing, including a loss of smell or altered taste ^27,45,46^. While anosmia has been detected in rodent models of SARS-CoV-2 infection ^28,29^, the present study is the first to demonstrate that adult RMs exhibit altered taste and smell after mild SARS-CoV-2 infection. At approximately 2.5 and 4 months after infection, RMs exhibited a decreased preference for the foods they most highly preferred at baseline. Additionally, at 2.5 months RMs had an increased preference for foods with the strongest flavors. While losses of smell and taste are most common during the acute infection period, patients have reported persistent smell and taste dysfunction for up to 2-years later with chemosensory recovery occurring over time ^27^. Future studies should have longer follow-up of food preference in RMs after SARS-CoV-2 infection to identify recovery of chemosensory function. People with smell loss will often report eating more salty or spicy foods to compensate for their blunted sense of taste ^47–49^. The increased preference for the more strongly flavored foods at 2.5 months post-infection may suggest a similar compensatory strategy in RMs following mild COVID-19 infection. Importantly, alterations in food preference were strongly correlated with the early changes in cytokine and chemokine levels from baseline. For example, greater increases in plasma IL6 during peak viremia corresponded with greater decreases in highly preferred foods after SARS-CoV-2 infection. In contrast, greater increases in plasma TARC and IL15 correspond with food preference closer to baseline. Taken together these results suggest a link between inflammatory and immune response to mild COVID-19 infection and chemosensory alterations.

Cognitive impairment or “brain fog” is highly common among PASC patients and has been reported up to 2 years post-infection in some cases ^12,30,31^. Brain fog symptoms can include a lack of focus or attention, forgetfulness, difficulty following directions or concentrating, decreased cognitive flexibility, and lack of inhibitory control ^50^. The current study revealed that adult RMs exhibit a deficit in cognitive flexibility at approximately 3 months after SARS-CoV-2 infection. Although short-term memory impairment has been demonstrated in a golden hamster model of PASC ^51^ this is, to our knowledge, the first study to demonstrate cognitive impairment after mild COVID-19 infection in the highly clinical relevant model of NHPs. The hamster PASC model indicated that earlier variants of SARS-CoV-2 result in greater memory impairment compared to later variants, which may explain the extent of cognitive deficits in our RMs infected with the Washington variant which was present in the US early during the pandemic. Interestingly, female RMs appeared to have greater deficits in cognitive flexibility as compared to males, which is consistent with PASC patient reports ^12,52^. The present study also found that changes in CSF levels of IL-4 and IL-12p70 positively correlates with cognitive flexibility deficits, suggesting that immune response to mild infection may explain the severity of PASC-like symptoms in RMs. Transcriptomic data from the hamster PASC model suggest long-term dysregulation in domapinergic and glutamateric signaling ^51^. Future studies should investigate whether similar neurotransmission changes are occurring in our RMs model.

Fatigue, post exertional malaise and sleep disturbances are commonly reported in PASC and other infection associated chronic illnesses ^1,30^. Although overall daytime activity was similar to baseline, RMs exhibited increased nighttime activity and higher fragmentation of circadian rhythm features, including intradaily variability and intradaily stability. These alterations in nighttime activity replicate a previous finding of increased sleep disturbances in both obese and lean RMs after SARS-CoV-2 infection ^18^. The lack of any differences in daytime activity or fatigue may be due to housing limitations that are less conducive to exercise exertion in RMs. Future studies should incorporate larger housing or exercise/play caging to better probe symptoms of post exertional malaise in RM models of PASC.

Previous studies have reported increased neuroinflammation, neuronal injury, and microhemorrhages in the brains of NHPs after mild SARS-CoV-2 infection ^21,24,53^, although several included relatively short follow-up of 7 to 49 dpi. The current study in adult RMs revealed increased neuroinflammation detected up to 135 days after SARS-CoV-2 infection. Importantly, increased microglia detected in the insula, a deep brain area important for sensory processing and cognitive function (reward processing and decision making), corresponded with alteration in sleep and cognitive flexibility. These areas are also integral to the sympathetic and parasympathetic nervous system which demonstrated alterations after SARS-CoV-2 infection in females. Together these data suggest that even in the absence of severe disease, SARS-CoV-2 infection can result in long-term neuroinflammation that may explain persistent behavioral and autonomic nervous system changes after recovering from the initial infection.

The current study is not without its limitations. First, and most importantly, we were only able to characterize a limited set of behavioral changes after recovery from acute SARs-CoV-2 infection, thus the current study was not designed to probe multiple aspects of neurological and cognitive function after mild COVID-19 illness. However, this study has established the safety and feasibility of creating an animal model to study the long-term neurobehavioral consequences after recovery from a biosafety level 3 virus (i.e. SARS-CoV-2), therefore future studies should be able to incorporate more extensive assessments. Second, behavioral assessments were restricted to cage-side testing which did not enable us to probe core symptoms of infection-associated chronic illness, including fatigue and post exertional malaise symptoms. Finally, animals were only studied for 4.5 months post-infection. Although this longitudinal follow-up is longer than most animal models of SARS-CoV-2 infection, future studies should examine neurocognitive changes for years post-infection to better probe persistence or potential recovery of function.

The large number of people infected during the COVID-19 pandemic increased our awareness of infection associated chronic illness, due to the considerable number of patients experiencing recurring symptoms for weeks or months post-recovery. Yet, there is still a considerable gap in our understanding of how mild COVID-19 illness results in persistent and debilitating chronic illness symptoms of PASC. Our study demonstrated that SARS-CoV-2 infected RMs recapitulate several common PASC symptoms and provides a causative link between early inflammatory response and long-term neurocognitive changes, making RMs an ideal animal model to investigate potential treatments for patients.

## Methods

### Animals:

Ten adult Indian rhesus macaques (RMs; *Macaca mulatta*), 5 females and 5 males, were included in this study. Viral loads were measured at multiple time points after infection in nasal and throat swabs and in BAL fluids. Three RMs (one female and 2 males) were necropsied at 45dpi to investigate potential viral reservoirs in other tissues, while the remaining seven RMs were used for longitudinal neurocognitive assessments and necropsied between 134 -150 dpi. All animals were from a specific pathogen-free colony (negative for Herpes B, SIV, SRV, and STLV1), had not been previously used for SARS-CoV-2 infectious disease or vaccine studies, and did not have any clinical signs of infection prior to the study. Animals were fed primate chow (Purina Primate Chow, Old World Monkey formulation), oranges, and vegetable enrichment daily with water provided ad libitum. All housing was indoors on a 12 h light–dark cycle. Animals were housed in an ABSL-3 (Animal Biosafety Level 3) at the Emory National Primate Research Center (ENPRC) during SARS-CoV-2 infection. During the post-infection/recovery stage, animals were housed in an ABSL-2+ (Animal Biosafety Level 2 enhanced) building until the end of the study.

### SARS-CoV-2 infection and sample collections:

RMs were infected with 1.1×10^6^ plaque forming units (PFU) SARS-CoV-2 strain 2019-noCoV/USA-WA1/2020 via both the intranasal (1 mL) and intratracheal (1 mL) routes concurrently. The SARS-CoV2 viral stock used in RM experiments (USA-WA/2020 strain) was obtained from Tulane University. Longitudinal tissue collections of peripheral blood (PB); bronchoalveolar lavage (BAL); nasal and pharyngeal mucosal swabs (SWAB); cerebrospinal fluid (CSF) evaluated for the study are showed in Supplementary Figure 1. In addition to the collections listed above, at necropsy the following tissues were processed for mononuclear cells: nasopharynx, caudal (lower) lung, hilar lymph node (LN), axillary LN, mesenteric LN, colonic LN, jejunum, ileum, frontal cortex, cerebellum, basal ganglia, insula, heart, and spleen.

### Cytokine and Chemokine assays:

Plasma concentrations of inflammatory markers were assayed by the Emory Multiplexed Immunoassay Core (Emory University, Atlanta, GA) or at Tulane National Biomedical Research Center using a multiplex electrochemiluminescence immunoassay kit from Meso Scale Discovery (Rockville, MD) following the manufacturer’s protocols. BAL, EDTA plasma, and CSF were assayed using the V-PLEX NHP Cytokine 24-Plex (Catalog #K15058D) for interleukins-6, -2, -5, -7, -8, -10, -12, -15, -16, -17A, and -1B, tumor necrosis factor-beta (TNF-β), granulocyte macrophage colony stimulating factor (GM-CSF), Eotaxin, Eotaxin-3, interferon gamma (IFN- γ), chemokines CXCL-10/IP-10, CCL2/MCP-1, CCL3/MIP-1α, CCL4/MIP-1β, CCL13/MCP-4, CCL17/TARC, CCL22/MDC, and vascular endothelial growth factor-A (VEGF-A). BAL, plasma, and CSF were also assayed using the Mesoscale Discovery U-plex Custom Biomarker (Catalog #K15068M) for human fractalkine, human G-CSF, human interleukins -1RA, -18, and -23, human M-CSF, human MIF, human MIP-3β, human TRAIL, NHP MIP-3α, NHP interleukins -4, 12p70, and -13. Serum was assayed for kyneurenine and tryptophan by Quantall LLC (Mountain View, CA) using liquid chromatography–mass spectrometry (LC-MS), detailed description of the assay can be found in the Supplementary Materials.

### Histopathology and Immunohistochemistry (IHC):

Brain samples were collected from both uninfected and SARS-CoV-2-infected macaques during postmortem examination and at multiple designated timepoints. The tissues were fixed in 10% neutral buffered formalin (NBF), processed with a tissue processor, embedded in paraffin, and sectioned serially at 5 μm thickness. Subsequently, the sections were stained with hematoxylin and eosin (H&E). For immunohistochemistry (IHC), unstained brain sections were evaluated using either a SARS-CoV-2 (COVID-19) nucleocapsid (GenTex [GT135357] 1:1000), an anti-IBA1 (Abcam [ab178846] 1:2000), or an anti-CD3 epsilon (Abcam [ab16669] 1:200) antibody, with an automated Leica Bond RX platform (Leica Biosystems). Tissue sections were dewaxed with Bond Dewaxing Solution (Leica) at 72°C for 30 min, rehydrated with graded alcohol washes, and 1X Leica bond wash. Heat-induced epitope retrieval (HIER) was performed using Epitope Retrieval Solution 2 (Leica), heated to 100°C for 20 minutes. IBA1 antigen positive cells were quantified by counting and averaging cells in ten 20X high-power fields for each animal. Samples were evaluated by a board-certified veterinary pathologist using an Olympus bright-field microscope (BX53) and images were captured using a microscope-mounted Olympus digital camera (DP27).

### Neurocognitive and Physiological Assessments:

To maintain BSL2+ biosafety levels, all testing was adapted to be conducted in the animal housing room using a cage side version of a Wisconsin general testing apparatus. To reduce variability between testing during the baseline (pre-infection) and recovery (post-infection) stages, the animals were housed in the same building, the same three experimenters conducted the testing (SF, RR, JR) and wore the same BSL2+ PPE during both stages.

### Food Preference Task:

Each animals’ preference for food and nonfood items were assessed before and after SARS-CoV2 infection (77, 119 dpi). An expanded version of the Food Preference task was used, which included 11 different categories of food and nonfood Items for a total of 22 items; pome fruit (apple, pear), citrus (red grapefruit, lemon), root vegetable (carrot, radish), cruciferous (broccoli, cauliflower), allium (onion, garlic), orange bell pepper, yellow squash, salty (peanut, potato chip), chocolate (red mini-M&M, coco puff cereal), protein (boiled egg, beefy dog food), yellow paper, yellow yarn, cork, and rubber glove ^54^. Proteins and nonfood items were included as previous data indicated that brain lesions result in abnormally high preference for such items that are typically rejected by normal rhesus macaques (^54–56^).

For each trial, two equal sized items (two different foods, two different nonfood, or a food vs nonfood) were placed in each of the lateral food wells according to a pseudo-random schedule and the animal was given a maximum of 60 s to choose one, both or neither item. Over the five days of testing, animals received every possible pairing twice (once in each of the two left-right configurations). Experimenters scored which item the animal selected to retrieve first. If neither item was chosen in 60 s, the trial was scored as a balk. To control for any temporal biases in the testing procedures or animals’ motivation, all testing occurred prior to feeding daily chow (between ~10:00 and 14:00 h) and the testing order was generated randomly, but constant across the pre- and post-infection phases. Preference for food and nonfood items was calculated in terms of a percentage: (thetotalnumberoftimesafoodwasselectedfirst/thetotalnumberoffoodpresentations)X100.

### Object Retrieval Detour Task:

The Detour task ^32,33^ was assessed at baseline and 85 dpi. The task apparatus consisted of a small transparent optical illusion box (5” × 5” × 4”) opened on only one side and fixed on a tray. The box could be rotated so that its open side would be presented on the right or left with a reward placed in the center of the box. Subjects were allowed unlimited time to retrieve the reward as long as they continued attempting to respond. If no responses occurred within a maximum of 3 minutes, the trial was terminated and scored as a “failure.” However, none of the animals had “failures” scored during the baseline or post-infection stage of testing. Training consisted of 7 days as follows: animals were first habituated to the apparatus and quickly learned that retrieving the reward required inhibiting reaching directly in front and instead detouring around the barrier to the open side, which was presented on either the right or the left. Following this brief training, animals underwent four days of testing comprising 32 trials per session. Each trial required the animal to negotiate a detour on one side for 5–7 consecutive attempts, after which the open side switched, requiring 5–7 consecutive detours in the opposite direction (e.g., box opening on the left, left, left, left, left, then box opening on the right, right, right, right, right). These repetitive sequences increased the likelihood of perseverative responses, defined as successive repetitions of a previously rewarded reach. The reward was always placed in the center of the box and sessions were deemed “Easy” or “Difficult” based on the number of reversals. Several measures of performance were scored including perseverative reaches, barrier reaches, latency to retrieve the reward, hand preference, and side bias. Side bias correction was applied to the data before analysis for perseverative reaches across all trials, perseverative reaches during reversal trials only, and latency to retrieve the reward.

### Activity and Sleep Measures:

To monitor the activity patterns, animals wore an accelerometer (Actical, Respironics Inc., Murrysville, PA) attached to a primate collar. Care was taken to ensure that the collars did not restrict the animal’s respiration, feeding, or daily activity. Actical devices were programed to record data at 1 second epochs and were replaced approximately every 2 weeks during sedated veterinary accesses to monitor for viral clearance. Activity data from the 24hr period immediately following a veterinary sedation was excluded from analysis. Additionally, the first week of activity data was excluded from analysis because it was considered a time when the animals were being acclimatized to collars and accelerometers. The data from the acticals was downloaded using the Actiware algorithm software (Respironics). Overall activity during daytime and nighttime hours was analyzed according to the 12 hr light cycle in the animal housing room (e.g. lights-on between 0700–1659 hour). Non-parametric circadian rhythm features were calculated including intradaily variability (IV), intradaily stability (IS), activity counts of the most active 10-hour period (M10), activity accounts of the least active 5-hour period (L5), and relative amplitude (RA) ^57^. Although accelerometers do not provide a direct measure of sleep, “sleep” states are inferred by the absence of activity and “awake” states are inferred by threshold activity levels as defined below. “Sleep” was defined on a weighted sliding average with a threshold optimized for night-time activity for nonhuman primates (the threshold for determining the onset of sleep was set to 14, instead of the standard setting of 20 ^58,59^) and sensitive to detect night-time sleep disruption. The number of bouts of uninterrupted sleep and their average duration was calculated. The latency to fall asleep was defined as time between lights off and the beginning of the first ≥3 min period of uninterrupted sleep.

### Heart rate Variability (HRV):

Serial 6-lead EKGs (using limb leads) were captured for 5 consecutive minutes while subjects were under anesthesia (General Electric AMX 4, set to Goldberger I mode) and output as PDF files at Gain 10 mm/mV and Feed 25 mm/s, recorded at 500 Hz sampling frequency. The EKG data was concatenated across PDF pages to reconstruct the complete time and voltage sequence for the EKG session, for each lead. This time and voltage data was assessed for heart rate variability (HRV) parameters in MATLAB using the PhysioNet Cardiovascular Signal Toolbox ^60^. Briefly, this well-characterized toolbox assesses EKGs using rolling 200 second-long windows for each lead to calculate output parameters, which were then reduced to median values for each output parameter for the session. The EKG lead with the most reliable lead signal, as determined by the highest average (SQI), was deemed the “best” lead and used for comparisons of other output parameters. Standard deviation of the normal to normal interval (SDNN) and the root mean square of the differences in successive R-R interval (RMSSD) were used as the most relevant measures of HRV over this timeframe.

### Statistical Analyses:

Food Preference Task data were used to assess potential changes in taste and smell. The most highly preferred foods, strongly flavored / least preferred, and nonfoods were analyzed separately using a Linear Mixed Models (LMM) with Time post-infection (baseline, 77dpi, 119dpi) and Sex (female, male) as fixed factors and individual animal as a random factor.

Potential changes in cognitive flexibility were examined using total perseverations, reversal perseverations, and latency data from the Detour Task. Data were analyzed using a LMM with Time post-infection (baseline, 85dpi), Trial Type (easy, difficult), and Sex (female, male) as fixed factors and individual animal as a random factor.

Actigraphy data was examined for changes in overall activity at daytime or nighttime, circadian rhythmicity (IS, IV, M10, L5, relative amplitude), sleep latency, number and duration of sleep bouts. Data were analyzed using a LMM with Time post-infection (baseline, 1–19 weeks post infection [wpi]), and Sex (female, male) as fixed factors and individual animal as a random factor.

Heart rate variability, as measured by EKG, was examined for changes in autonomic function after SARS-CoV-2 infection. Data were analyzed using a LMM with Time post-infection (baseline, ~110, ~135 dpi) and Sex (female, male) as fixed factors and individual animal as a random factor.

All behavioral results were graphed as a difference from baseline to reflect the changes within and between animals more easily (tables of raw data can be found in the supplemental materials). Difference scores were also used for correlations between behavioral results and peak viral load or inflammatory markers. GraphPad Prism 10.01 (GraphPad Software) was used for all graphs and behavioral data was analyzed using SPSS 29 for Windows (IBM), significance was set at p < 0.05.

## Supplementary Material

Supplementary Files

This is a list of supplementary files associated with this preprint. Click to download.

• NHPPASCSupplementaryfigures112125.pdf
